# Designing Nursing Policy Intelligence for 2040: Japan as a Leading Case

**DOI:** 10.1111/inr.70215

**Published:** 2026-08-23

**Authors:** Kazumi Kubota

**Affiliations:** ^1^ Shimonoseki City University Yamaguchi Japan; ^2^ The University of Tokyo Hospital Tokyo Japan; ^3^ Health and Global Policy Institute Tokyo Japan; ^4^ National Center for Child Health and Development Setagaya Japan

**Keywords:** decision‐grade nursing policy intelligence, governance, health policy, nursing workforce, policy capacity, workforce planning

## Abstract

**Aim:**

To propose design requirements for decision‐grade nursing policy intelligence that can translate nursing workforce and professional development signals into implementable policy action for the 2040 horizon.

**Background:**

Health systems planning for 2040 face population ageing, fiscal constraint and rising care complexity. The central problem is not only estimating nursing supply but converting signals on workforce flows, career development, workload, supervision capacity, retention and service outcomes into timely, legitimate decisions. Japan is used as a leading case because these challenges are already visible.

**Sources of Evidence:**

This non‐empirical policy analysis used a structured, purposive source‐selection strategy. Searches of two bibliographic nursing and health databases for literature published from January 2014 to 5 May 2026 were supplemented by targeted searches of international and Japanese policy repositories and citation chasing. Sources were charted by evidence type, policy focus, geographic scope and policy relevance.

**Discussion:**

The analysis argues that nurses’ policy engagement is necessary but insufficient unless health systems also build a governed translation capability. Using career development and continuing professional development as a practical use case, the paper proposes seven design requirements: co‐production through boundary roles; alignment with decision windows; public data stewardship; transparent scenario modelling; rapid‐cycle products; implementation feedback; and safeguards.

**Conclusion:**

Decision‐grade nursing policy intelligence offers an intelligence‐to‐action pathway for workforce reform under demographic constraint. Japan provides a useful case for specifying adaptable governance conditions.

**Implications for nursing:**

Intelligence‐to‐action pathways can help nurse managers link professional development, deployment, supervision capacity, workload sustainability and retention.

**Implications for nursing policy:**

Policymakers and system leaders should treat nursing policy intelligence as shared infrastructure for aligning workforce signals with budget, regulatory and implementation decisions.

## 1 Aim

This paper proposes design requirements for decision‐grade nursing policy intelligence that strengthens the nursing profession's capacity to translate workforce and professional development signals into implementable reforms by 2040.

## 2 Background

Health systems increasingly use long‐term horizons such as 2035 and 2040 to frame reform under population ageing, fiscal constraint and rising care complexity (OECD 2023; World Health Organization 2025). These horizons are not simply forecasts. They are practical planning frames that shape when budgets are set, regulations are revised and workforce plans are recalibrated. Recent workforce‐planning scholarship also argues that planning should move beyond linear forecasting and be driven by strategy, policy choices and normative futures (Rees et al. 2025). A central problem for nursing policy is that many systems collect workforce information but lack a reliable mechanism for translating it into timely and implementable decisions.

Japan's “2040 cliff” provides a particularly instructive leading case. Japan is not presented here as a model to be copied directly, but as an early and visible case of pressures that many ageing societies will face: a shrinking working‐age population, rising care demand, fiscal constraint and growing dependence on effective nursing workforce policy. These conditions make the cost of slow policy cycles more apparent and help reveal governance bottlenecks that may otherwise remain less visible until later in other systems. The 2040 horizon is also becoming an explicit professional policy horizon in Japan. The Japanese Nursing Association's *Future Vision of Nursing 2040* frames nursing as central to sustaining human life, living and dignity in a changing society (Japanese Nursing Association 2025). This makes Japan useful not only as a demographic case but also as a policy case for examining how nursing visions, workforce signals and decision‐making can be connected under constraint.

In this paper, workforce signals refer to information that should alert policy actors to emerging risks or opportunities for action. These include workforce entries and exits, inactive licences, continuing professional development participation and completion, workload, supervision capacity, retention and turnover, role utilisation, and service quality and safety indicators. Such signals become policy‐relevant only when they are interpreted, prioritised and connected to decision windows.

In this context, sustaining nursing cannot be reduced to supply pipelines alone. Policy must also make nursing careers workable and worth sustaining over a working life, including credible progression and continuing professional development that are feasible within work‐design constraints and organisational capacity. Workforce policy therefore depends on whether systems can routinely convert signals, about retention, progression, skill development, and service outcomes, into decisions that are timely, legitimate and implementable. The argument advanced here is that many systems struggle because the capability to translate evidence into decision‐ready policy options is weak or fragmented.

**FIGURE 1:**
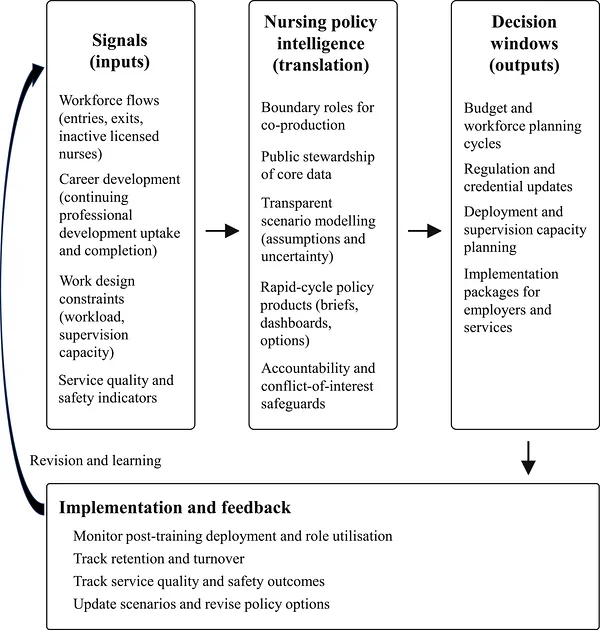
Nursing policy intelligence for the 2040 horizon: an intelligence‐to‐action framework. Workforce, career‐development, work‐design and service‐quality signals are translated into decision‐ready policy products aligned with budget, regulatory, workforce‐planning and implementation decision windows. Implementation feedback is used to update assumptions, revise scenarios and adjust policy options.

In this paper, nursing policy intelligence is defined as an end‐to‐end capability to curate workforce and professional development signals, translate them into decision‐ready policy products aligned with budget and regulatory cycles, and learn from implementation through feedback and revision (World Health Organization 2016). This definition emphasises policy use: intelligence is valuable when it helps decision‐makers compare feasible options, understand assumptions and uncertainty, and revise action as implementation evidence accumulates. Figure 1 summarises this intelligence‐to‐action logic.

## 3 Sources of Evidence

This article is a non‐empirical policy analysis using a structured, transparent and purposive source‐selection strategy. It was not designed as a systematic or scoping review and did not aim to exhaustively identify all eligible studies or estimate pooled effects. The purpose of the source search was to identify review‐level evidence, governance analyses and policy documents capable of informing design requirements for decision‐grade nursing policy intelligence.

Bibliographic searches were conducted in PubMed/MEDLINE and CINAHL for literature published from January 2014 to 5 May 2026. Three targeted search strategies were used. The first focused on nurses’ policy engagement and health policy development. The second focused on nursing and health workforce governance, workforce planning, stewardship and accountability. The third focused on continuing professional development, career development, implementation, retention, data analytics and decision‐making. Searches used title/abstract terms and review‐type terms where appropriate. A supplementary Japan‐specific search combined Japan, nursing, continuing professional development, continuing education, career development, specified medical acts, designated clinical practice, training and workforce terms.

Publicly available policy and professional documents were identified through targeted searches of international and national repositories and websites, including the World Health Organization, the Organisation for Economic Co‐operation and Development, the International Council of Nurses, the Japanese Nursing Association, the Ministry of Health, Labour and Welfare of Japan, and Health and Global Policy Institute/Japan Health Policy NOW. International Council of Nurses sources, including leadership‐capacity resources such as the Global Nursing Leadership Institute, were consulted to situate the proposed boundary‐role and policy‐capacity requirements within existing international nursing infrastructure (International Council of Nurses n.d.). Japan Health Policy NOW was consulted as a bilingual contextual source on Japanese health policy (Health and Global Policy Institute n.d.). Citation chasing from included reviews and policy documents was also used to identify additional policy‐relevant sources.

Sources were included when they addressed at least one of the following domains: nurses’ engagement in policy development; health or nursing workforce governance; workforce planning; continuing professional development or career development; analytic capacity or decision‐making in nursing management; economic value or return on investment in nursing; or implementation feedback relevant to workforce reform. Japan‐specific sources were included when they provided policy‐relevant examples of continuing professional development, role development, workforce planning, specialised nursing capacity or health‐system governance relevant to the 2040 horizon. Sources were excluded when they focused only on clinical education content without workforce or policy relevance, did not address nursing or health workforce governance, were not publicly accessible, or were not available in English or Japanese.

The included sources were charted by evidence type, policy focus, geographic scope and contribution to the proposed design requirements. Review‐level evidence was used to identify recurring conditions that enable or constrain nurses’ policy engagement, career development, workforce governance and implementation. Policy documents were used to frame the 2035–2040 planning horizon and the governance expectations for workforce planning. Japan‐specific sources were used illustratively, not as an exhaustive national policy mapping. The synthesis was interpretive and policy‐oriented, comparing evidence across domains to identify governance conditions that could connect workforce and professional development signals to decision windows and implementation feedback.

The updated searches identified recent policy‐relevant sources directly related to the manuscript's central argument, including work on strategy‐driven health workforce planning, data‐to‐intelligence processes, workforce data standardisation, nurses’ continuing professional development and Japan‐specific specialised nursing capacity (Lyn et al. 2026; Matandela et al. 2026; Morioka et al. 2026; Rees et al. 2025; Simkin et al. 2025). Recent policy documents, including WHO's *State of the World's Nursing* 2025 and the Japanese Nursing Association's *Future Vision of Nursing 2040*, were also added, where they directly informed the 2040 workforce‐policy framing (Japanese Nursing Association 2025; World Health Organization 2025).

## 4 Discussion

This discussion addresses the paper's aim by asking what kind of policy capability is needed to translate nursing workforce and professional development signals into implementable reform for the 2040 horizon. Five linked arguments are developed: policy engagement must be connected to translation capacity; career development and continuing professional development provide a practical test case; nursing policy intelligence requires explicit design requirements; decision‐grade products must be aligned with decision windows; and implementation feedback is essential for legitimacy and learning.

### 4.1 From Policy Engagement to Translation Capacity

Review‐level evidence indicates that nurses’ engagement in policy development is shaped by modifiable conditions such as leadership development, organisational support, protected time and meaningful access to decision‐making processes (Banisalman et al. 2026; Smith et al. 2025). Hospital‐level policy involvement, for example, is facilitated when nurses have institutional support and credible channels to influence decisions, and is constrained when participation is symbolic or disconnected from decision authority (Banisalman et al. 2026). Internationally, nurses’ roles and barriers are also shaped by wider governance arrangements and resourcing environments, particularly in community settings and low‐ and middle‐income countries, where workforce constraints and system fragmentation can limit the translation of nursing knowledge into policy (Touzami et al. 2025).

These findings support the need to strengthen nurses’ participation in policy. However, participation alone is unlikely to produce durable reform if policy systems lack mechanisms to convert nursing knowledge and workforce signals into decision‐ready options. This distinction is important for the 2040 horizon. Long‐term workforce pressures require decisions about budgets, regulation, education capacity, role design and deployment. If nursing input enters policy processes only episodically, or after key decisions have already been made, engagement may remain symbolic. Nursing policy intelligence therefore needs to function as a translation capability: It should connect frontline and workforce signals to the timing, language and constraints of real policy decisions.

### 4.2 Career Development and Continuing Professional Development as a Test Case

Career development and continuing professional development provide a concrete use case because they connect workforce sustainability, retention, service quality and professional legitimacy. A system may offer continuing professional development programmes but still fail to achieve scalable progression if nurses cannot participate because of workload, staffing shortages, lack of protected time or limited supervision capacity.

Recent review evidence on nurses’ continuing professional development suggests that engagement is shaped not only by programme availability but also by perceived value, learning agency, workplace conditions and opportunities to apply learning to practice (Matandela et al. 2026). This supports the argument that continuing professional development should be governed as part of work design, deployment and retention, rather than treated as a stand‐alone educational activity.

Japanese examples illustrate this problem. The Japanese Nursing Association describes continuing professional development courses intended to strengthen knowledge and technical skills across practice, administration and education (Japanese Nursing Association n.d.). Yet barriers to continuing education and continuing professional development have been reported among occupational health nurses in Japan, including constraints related to work and organisational support (Mizuno‐Lewis et al. 2014). Structured training linked to designated clinical practices in home care has also been surveyed nationally, suggesting that formal role‐development systems can exist while awareness, uptake and organisational readiness vary (Tomita et al. 2021).

Recent Japanese evidence on certified nurses and certified nurse specialists further shows that specialised nursing capacity has been geographically uneven, with some prefectures maintaining persistently low densities despite overall growth (Morioka et al. 2026). This reinforces the need to link career development to deployment and workforce planning rather than treating certification only as an individual professional achievement. Together, these examples show why the policy challenge is not simply ‘creating a programme’ but governing the pathway from professional development to deployment, capability utilisation and retention at scale.

### 4.3 Seven Design Requirements for Nursing Policy Intelligence

The synthesis suggests seven design requirements for decision‐grade nursing policy intelligence.

First, co‐production through boundary roles is needed so that nurses, managers, educators, policymakers, employers, regulators and service users can jointly define policy problems and feasible options. Boundary roles are important because workforce intelligence sits between professional knowledge, management routines, regulatory authority and political decision‐making.

Second, intelligence products should be aligned with decision windows, including budget cycles, regulatory reviews, credentialing processes, workforce planning timetables and service capacity planning. Evidence that arrives after a decision window has closed may be technically sound but practically weak.

Third, public stewardship of core workforce data is needed to maintain stable definitions and trusted access arrangements for indicators such as progression, deployment, retention and role utilisation. Workforce intelligence depends on data that are comparable over time and credible across stakeholders.

Fourth, scenario modelling should be transparent, with assumptions and uncertainty made visible rather than hidden in technical outputs. This is particularly important when capacity constraints such as training slots, supervision availability, workload and fiscal ceilings determine what is feasible in practice.

Fifth, analytic work should be translated into rapid‐cycle policy products, such as briefs, dashboards and options papers that specify choices, constraints, implementation burden and monitoring plans. The purpose is not to produce more reports but to make evidence usable within the time frame of decisions.

Sixth, implementation feedback loops should track whether trained nurses are deployed into intended roles, whether supervision capacity is sufficient, and whether retention and service‐quality outcomes change. Policy intelligence should therefore continue after a policy is announced.

Seventh, safeguards for transparency and conflicts of interest are needed because workforce reform can affect credentialing, training markets, employer incentives, professional status and resource allocation. Legitimate policy intelligence requires clarity about who defines the problem, who controls the data, who benefits from proposed options and how uncertainty is communicated.

### 4.4 Aligning Intelligence Products With Decision Windows

For policy intelligence to be useful, it must be time‐aware and product‐oriented. Evidence on data analytics and administrative decision‐making in nursing management suggests that data have value when they are translated into decisions and routines, rather than remaining detached technical artefacts (Darach et al. 2025). The same principle applies at the system level. A workforce dashboard released after a budget decision, or a professional development report disconnected from regulation and deployment planning, is unlikely to change policy.

The point is not simply to collect more data. Recent health workforce‐planning work emphasises the movement from data to intelligence, in which raw data and indicators are transformed into usable, decision‐relevant information for planners and decision‐makers (Simkin et al. 2025). For nursing, this means that dashboards, continuing professional development reports and workforce analyses should be designed around the decisions they are intended to inform.

Decision‐grade products should therefore be designed around the decisions they are meant to inform. For example, a briefing for a budget cycle should specify the cost and capacity implications of expanding continuing professional development access. A regulatory options paper should identify how credentialing changes would affect supervision requirements and role utilisation. A workforce‐planning dashboard should show not only headcounts but flows into and out of practice, inactive licences, training uptake, post‐training deployment and retention.

Public stewardship of core workforce data is especially important because workforce intelligence depends on stable definitions and trusted data‐sharing arrangements. Recent work on health workforce data standardisation shows that legal, regulatory and governance levers may be needed to support harmonised data collection and sharing across organisations (Lyn et al. 2026). Without such arrangements, indicators such as progression, deployment, retention and role utilisation are difficult to compare or govern.

### 4.5 Feedback, Accountability and Legitimacy

Workforce governance depends on how evidence, authority and accountability are organised across multiple actors (Lim and Lin 2021). Historical evidence from health workforce governance also shows that episodic reform can drift from its original intent when implementation realities are not monitored and fed back into policy revision (Van Ryneveld et al. 2020). For nursing, this means that policy intelligence should not end with recommendations. It should continue through implementation monitoring and revision.

Recent global nursing workforce evidence reinforces this point. *WHO's State of the World's Nursing* 2025 emphasises that nursing workforce policy must connect education, jobs, leadership and service delivery rather than treating workforce supply as a stand‐alone problem (World Health Organization 2025). In the 2040 context, this means that professional development and career progression should be linked to deployment, retention, supervision capacity and service outcomes.

Economic evidence also matters because decision‐makers often need to consider costs, benefits and trade‐offs in explicit terms (Yakusheva et al. 2025). Presenting nursing investments only as professional aspirations or staffing counts may be insufficient under fiscal constraint. A decision‐grade approach should make assumptions transparent, allow contestation and show how different options may affect workforce sustainability, service quality and organisational value. This does not reduce nursing to economics; rather, it helps nursing policy claims enter decision spaces where resources, timing and accountability are negotiated.

### 4.6 Limitations

This article is a non‐empirical policy analysis and does not report primary research, causal inference or a formal systematic review. Although a structured source‐selection process was used, the synthesis remains interpretive, and different source selections could lead to additional emphases or alternative design requirements.

Japan is used as a leading case to illustrate governance mechanisms and bottlenecks, not as a model that can be transferred directly to other countries. Institutional arrangements, professional structures, labour‐market conditions and data‐governance systems differ across settings. Transferability therefore requires local adaptation, particularly regarding who can steward workforce data, where boundary roles should be located and how decision windows operate.

Finally, the proposed intelligence‐to‐action framework has not been empirically evaluated. Future work should examine whether boundary roles, decision‐window alignment, minimum workforce datasets and implementation feedback loops improve the timeliness, legitimacy and effects of nursing workforce‐policy decisions.

## 5 Conclusion

The 2040 horizon requires nursing workforce policy to move beyond supply estimates and episodic engagement. This policy analysis proposes decision‐grade nursing policy intelligence as an intelligence‐to‐action capability that connects workforce and career‐development signals to budget, regulatory, workforce‐planning and implementation decisions. Japan offers a useful leading case because its demographic pressures and explicit nursing 2040 agenda make translation and governance bottlenecks visible earlier than in many other health systems. Building this capability can help policymakers and nursing leaders design workforce reforms that are more timely, legitimate, feasible and responsive to implementation learning.

## 6 Implications for Nursing Practice

For nurses and nurse managers, an intelligence‐to‐action pathway can make professional development more workable in daily practice by treating it as support system work rather than as an individual responsibility alone. Evidence on continuing professional development indicates that engagement is shaped by perceived value, learning agency, workplace conditions and opportunities to apply learning in practice, while Japanese evidence also shows that workload, time and organisational support can constrain participation even when programmes exist (Matandela et al. 2026; Mizuno‐Lewis et al. 2014). In practice, this means that nurse managers need usable intelligence on where staffing pressure, lack of protected time, limited supervision capacity or unclear deployment pathways prevent nurses from taking up and using professional development opportunities.

The framework also links professional development to role utilisation, career progression and retention. Monitoring whether nurses who complete training are deployed into appropriate roles can help reduce frustration, make progression more visible and support retention. This is consistent with evidence that coherent career frameworks can support specialised practice and career sustainability, and with Japanese evidence showing that specialised nursing capacity may remain unevenly distributed without deliberate workforce planning (Morioka et al. 2026; Thennakoon et al. 2025). For practice leaders, the implication is practical: professional development plans should be considered alongside workload sustainability, supervision arrangements, deployment pathways and nurse‐sensitive service quality and safety indicators, rather than being planned as education activity alone (Darach et al. 2025; World Health Organization 2025).

## 7 Implications for Nursing Policy, Health Policy and Social Policy

Policymakers and system leaders should treat nursing policy intelligence as shared workforce infrastructure, not as a periodic report or a professional advocacy activity. Review‐level evidence shows that nurses’ policy engagement is enabled or constrained by leadership, organisational access and support, but engagement alone is unlikely to produce reform if decision windows, authority and accountability remain weak (Banisalman et al. 2026; Smith et al. 2025). Workforce governance literature similarly indicates that sustainable reform depends on how systems organise evidence, authority and accountability across multiple actors (Lim and Lin 2021; Van Ryneveld et al. 2020). The policy implication is that governments, regulators, employers, educators and professional bodies should establish boundary roles and routines that connect nursing practice knowledge to budget cycles, credentialing updates, workforce plans and service‐planning decisions.

A second implication is that workforce data stewardship should be treated as a governance function. Minimum datasets should link workforce flows, continuing professional development participation, post‐training deployment, role utilisation, retention and nurse‐sensitive service outcomes. Recent health workforce‐planning literature emphasises that data must be transformed into decision‐relevant intelligence, and that standardised workforce data may require legal, regulatory and governance levers for harmonised collection and sharing (Lyn et al. 2026; Simkin et al. 2025). Scenario modelling and policy products should therefore make assumptions, uncertainty, implementation burden and conflicts of interest visible before decisions are locked in. Under demographic and fiscal constraints, this kind of decision‐grade intelligence can help nursing workforce reform move from aspiration to implementable and accountable action, while also making the expected value and trade‐offs of nursing investments more visible to decision‐makers (Rees et al. 2025; Yakusheva et al. 2025).

## Author Contributions

Study design; identification and review of publicly available sources; synthesis and interpretation; manuscript writing; Critical revisions for important intellectual content.

## Conflicts of Interest

No conflict of interest has been declared by the author. The author has nothing to report.

## Ethical Considerations

This article is a non‐empirical policy analysis and review of publicly available literature and policy documents. No human participants were recruited, and no identifiable personal data were collected or analysed. Therefore, formal ethics committee approval and informed consent were not required.

## Funding

This research received no specific grant from any funding agency in the public, commercial or not‐for‐profit sectors. The author has nothing to report.

## AI Use Statement

Artificial intelligence tools were used to support drafting and language editing of this manuscript and to assist with formatting references. All outputs were reviewed, verified and edited by the author, who takes full responsibility for the content, including accuracy of statements and citations. No confidential or proprietary data were uploaded to AI tools.

## References

[inr70215-bib-0001] Banisalman, I. M. , J. Lanzillotta‐Rangeley , L. Warner , and C. R. Smith . 2026. “Nurses Involvement in Hospital Health Policy Development: Benefits and Barriers: An Integrative Review.” Western Journal of Nursing Research 48, no. 2: 194–205. 10.1177/01939459251387801. 41355292

[inr70215-bib-0002] Darach, N. , M. S. Kim , W. Wisesrith , and E. G. Collins . 2025. “Data Analytics and Administrative Decision‐Making in Nursing Management: A Systematic Review.” Journal of Nursing Management 2025: 4344147. 10.1155/jonm/4344147. 41235177 PMC12611473

[inr70215-bib-0003] Health and Global Policy Institute . n.d. Japan Health Policy NOW. Available at: https://japanhpn.org/en/home‐2/ (accessed 5 May 2026).

[inr70215-bib-0004] International Council of Nurses . n.d. Global Nursing Leadership Institute (GNLI). Available at: https://www.icn.ch/how‐we‐do‐it/icn‐leadership‐centre/global‐nursing‐leadership‐institutetm‐gnli (accessed 20 December 2025).

[inr70215-bib-0005] Japanese Nursing Association . n.d. Continuing Professional Development (CPD): CPD Courses. Available at: https://www.nurse.or.jp/english/activities/en_projects.html (accessed 5 May 2026).

[inr70215-bib-0006] Japanese Nursing Association . 2025. *Future Vision of Nursing 2040: Nursing Supports and Sustains Human Life, Living and Dignity*. Japanese Nursing Association, Tokyo. Available at: https://www.nurse.or.jp/english/activities/future_vision_of_nursing2040.html (accessed 5 May 2026).

[inr70215-bib-0007] Lim, M. Y. H. , and V. Lin . 2021. “Governance in Health Workforce: How Do We Improve on the Concept? A Network‐Based, Stakeholder‐Driven Approach.” Human Resources for Health 19, no. 1: 1. 10.1186/s12960-020-00545-0. 33388068 PMC7777277

[inr70215-bib-0008] Lyn, A. , K. Leslie , A. Sweetman , et al. 2026. “Standardizing Health Workforce Data in Canada: Legal and Regulatory Levers for Harmonized Collection and Sharing.” Healthcare Management Forum 39, no. 3: 255–262. 10.1177/08404704251403158. 41628399 PMC13039225

[inr70215-bib-0009] Matandela, M. , N. J. Muswede , and V. E. Matahela . 2026. “Understanding Nurses' Dispositions for Continuing Professional Development: A Scoping Review.” Nurse Education in Practice 93: 104834. 10.1016/j.nepr.2026.104834. 41985417

[inr70215-bib-0010] Mizuno‐Lewis, S. , K. Kono , D. R. Lewis , et al. 2014. “Barriers to Continuing Education and Continuing Professional Development Among Occupational Health Nurses in Japan.” Workplace Health & Safety 62, no. 5: 198–205. 10.1177/216507991406200504. 24806039

[inr70215-bib-0011] Morioka, N. , T. Tamaki , and K. Takahashi . 2026. “An Ecological Panel Analysis of Trends in the Geographic Disparities of the Certified Nurse and Certified Nurse Specialist in Japan From 1996 to 2022.” Nursing Reports 16, no. 1: 25. 10.3390/nursrep16010025. 41591122 PMC12844417

[inr70215-bib-0012] OECD . 2023. Health at a Glance 2023: OECD Indicators. OECD Publishing. Available at: https://www.oecd.org/health/health‐at‐a‐glance/ (accessed 5 May 2026).

[inr70215-bib-0013] Rees, G. H. , G. Willis , and C. Scotter . 2025. “Health Workforce Planning Should be Strategy or Policy‐Driven: From Linear Forecasts to Normative Futures.” Health Policy 161: 105440. 10.1016/j.healthpol.2025.105440. 40997530

[inr70215-bib-0015] Simkin, S. , C. Damba , B. Farah , et al. 2025. “From Data to Intelligence for Health Workforce Planning: Insights From Integrated Primary Care Workforce Planning in Toronto.” Healthcare Management Forum 38, no. _suppl1: S59–S64. 10.1177/08404704251365552. 41151076

[inr70215-bib-0016] Smith, L. , K. J. Roberts , B. Giambra , et al. 2025. “Nurses' engagement in Healthcare Policy Development: An Umbrella Review.” International Nursing Review 72, no. 4: e70109. 10.1111/inr.70109. 41021299

[inr70215-bib-0017] Thennakoon, S. , S. G. M. Ang , V. Traynor , and K. Strickland . 2025. “An Integrative Review of Specialised Nursing Career Frameworks to Develop a Nursing Career Framework for Registered Nurses Working in Aged Care.” Journal of Advanced Nursing 81, no. 8: 4447–4464. 10.1111/jan.16674. 39696940 PMC12271661

[inr70215-bib-0018] Tomita, M. , C. Sato , H. Suzuki , M. Kanako , and M. Watanabe . 2021. “Home‐Visit Nurses' Awareness of the Specified Medical Acts Training System in Home Care: A National Survey of Home‐Visit Nursing Stations.” Journal of Japan Academy of Nursing Science 41: 250–258. 10.5630/jans.41.250.

[inr70215-bib-0019] Touzami, S. , B. Bencharki , M. Jaafar , E.l H. Roussi , A. Barkat , and F. Z. Laamiri . 2025. “Community Health Nurses in Low‐ and Middle‐Income Countries: A Systematic Integrative Review of Roles, Barriers, and Improvement Perspectives.” International Nursing Review 72, no. 4: e70128. 10.1111/inr.70128. 41251397

[inr70215-bib-0020] Van Ryneveld, M. , H. Schneider , and U. Lehmann . 2020. “Looking Back to Look Forward: A Review of human Resources for Health Governance in South Africa From 1994 to 2018.” Human Resources for Health 18, no. 1: 92. 10.1186/s12960-020-00536-1. 33243260 PMC7689387

[inr70215-bib-0021] World Health Organization . 2016. Global Strategy on Human Resources for Health: Workforce 2030. WHO. Available at: https://www.who.int/publications/i/item/9789241511131 (accessed 5 May 2026).

[inr70215-bib-0022] World Health Organization . 2025. *State of the World's Nursing 2025: Investing in Education, Jobs, Leadership and Service Delivery*. WHO. Available at: https://www.who.int/publications/b/78362 (accessed 5 May 2026).

[inr70215-bib-0023] Yakusheva, O. , K. Lee , A. V. Fial , and M. E. Weiss . 2025. “Organizational Return on Investment in Nursing: A Systematic Review.” International Journal of Nursing Studies 170: 105146. 10.1016/j.ijnurstu.2025.105146. 40617154

